# Study on the metabolic process of phthalic acid driven proliferation of *Rhizoctonia solani*

**DOI:** 10.3389/fpls.2023.1266916

**Published:** 2023-10-11

**Authors:** Jidong Ju, Bingqian Zhou, Guohong Yang, Xinyu Fu, Xiao Wang, Lanping Guo, Wei Liu

**Affiliations:** ^1^ Key Laboratory for Applied Technology of Sophisticated Analytical Instruments of Shandong Province, Shandong Analysis and Test Center, Qilu University of Technology (Shandong Academy of Sciences), Jinan, China; ^2^ Key Laboratory for Natural Active Pharmaceutical Constituents Research in Universities of Shandong Province, School of Pharmaceutical Sciences, Qilu University of Technology (Shandong Academy of Sciences), Jinan, China; ^3^ Pharmaceutical Institute, Shandong University of Traditional Chinese Medicine, Jinan, China; ^4^ Chinese Medicine Resource Center, China Academy of Chinese Medical Sciences, Beijing, China

**Keywords:** phthalic acid, *Rhizoctonia solani*, widely targeted metabolomics, synergistic damage, metabolism network

## Abstract

**Introduction:**

Continuous cropping obstacle seriously affects the quality and yield of *Salvia miltiorrhiza*, and the synergistic effect of root exudates and rhizosphere pathogenic microorganisms may be an important cause of continuous cropping obstacle. This study aimed to explore the effects of representative organic acids on the growth and metabolism of specific microorganisms in the *S. miltiorrhiza* rhizosphere soil under continuous cropping, and clarify its mechanism.

**Methods:**

The effect of phthalic acid (PA) on the growth and metabolism of *Rhizoctonia solani* was evaluated by mycelial growth inhibition method. Ultra-high performance liquid chromatography and tandem mass spectrometry were used to identify the differential metabolites of *R. solani* induced by exogenous PA.

**Results:**

PA exerted a concentration-dependent effect on mycelial growth, biomass, intracellular polysaccharides con-tent, and total protein content in *R. solani*. A total of 1773 metabolites and 1040 differential metabolites were identified in the blank medium (CK), Fungi (CK + fungi), and PA-Fungi (CK + fungi + acid) groups. Kyoto Encyclopedia of Genes and Genomes (KEGG) enrichment analysis showed that the differential metabolites were mainly involved in the sugar, lipid, and protein metabolic pathways related to stable membrane structure and cell growth.

**Discussion:**

The proliferation and metabolism network of *R. solani* induced by PA was proposed, and the enhancement of sugar, lipid, and amino acid metabolism was presumed to be related to the active resistance of cells to organic acid stress. These results offer new in-sights into the effects of PA metabolism on promoting *R. solani* proliferation, and provide theoretical support for further optimizing the rhizosphere microecological environment of *Salvia miltiorrhiza* continuous cropping soil and reducing continuous cropping obstacle.

## 1 Introduction

*Salvia miltiorrhiza* (Danshen) is one of the most economically important medicinal plants used as a raw material for the production of cardiovascular drugs, human health foods, and cosmetics (Li et al., 2018; Sun et al., 2022). However, consecutive monoculture of *Salvia miltiorrhiza* results in disease aggravation, poor growth status, yield reduction, and quality deterioration (Liu et al., 2020a). Continuous cropping obstacles are mainly caused by three factors: imbalance in the rhizosphere microbial community structure, allelopathic autotoxicity, and deterioration of soil physical and chemical properties (Dong et al., 2018; Xin et al., 2019; Liu et al., 2021; Na et al., 2021; Tan et al., 2021). Increasing studies have shown that specific autotoxins from root exudates not only directly suppress plant growth, but also indirectly stimulate soil-borne pathogens, thus enhancing disease incidences (Fang et al., 2018; Guo et al., 2020; Chen et al., 2021; Fei et al., 2021). In addition, the synergistic effect of root exudates and rhizosphere pathogenic microorganisms may be an important cause of continuous cropping obstacles (Li et al., 2014; Wu et al., 2016). Therefore, there is an urgent need to elucidate the precise mechanisms of the allelopathy between autotoxins and pathogens, especially in the case of medicinal plant production.

Continuous monoculture can destroy the rhizosphere microbial community structure and soil health (Tang et al., 2015). Our previous studies have indicated that the fungi:bacteria ratio in the *S. miltiorrhiza* rhizosphere significantly increased with the in-crease in monoculture years, along with the presence of new pathogenic fungi such as *Rhizoctonia solani* (Liu et al., 2019). Furthermore, phthalic acid (PA) has been found to be the main allelopathic autotoxin in *S. miltiorrhiza* rhizosphere soil, and its content significantly increased after continuous cropping (Jiao et al., 2022). Wu et al. found that PA significantly stimulated mycotoxin production and pathogenicity-related hydrolase activity in Fusarium oxysporum (Wu et al., 2015). However, the effects of organic acids on the physiological characteristics and metabolic processes of *R. solani* are still unclear.

At present, research on the interaction mechanism between root exudates of medicinal plants and rhizosphere microorganisms mainly focuses on three aspects, namely, carbon and nitrogen sources and energy supply, induced chemotaxis effect, and soil physical and chemical properties (Bakker et al., 2018; Kuypers et al., 2018; Sasse et al., 2018; Chai and Schachtman, 2022). On the one hand, plant photosynthetic carbon can be absorbed by specific microorganisms in the rhizosphere (Hernandez et al., 2015; Cordovez et al., 2019), providing a dynamic source for a series of physiological activities such as proliferation and colonization, mycelial growth, and spore germination (Zhao et al., 2015; Zhalnina et al., 2018). On the other hand, organic acids secreted by plant roots can promote soil acidification and affect the proliferation and colonization of certain microorganisms (Liu et al., 2021; Na et al., 2021). At the same time, root exudates can selectively recruit growth-promoting or pathogenic fungi to the rhizosphere through chemical induction and microbial substrates, thereby affecting the growth and metabolism of the host (Turrà et al., 2015; Hu et al., 2018; Ding et al., 2020). Therefore, development of a technique that can determine and compare metabolites before and after co-culture of pathogenic fungi with auto-toxins can enhance our understanding of the synergistic effects of root exudates and soil pathogens on continuous cropping of plants.

In recent years, widely targeted metabolomics has become an effective method for qualitative and quantitative analyses of low molecular weight metabolites of microorganisms (Yang et al., 2019; Xiao et al., 2021; Zhao et al., 2021). This technology aims to explain the relationship and information flow between microorganisms and phenotypes resulting from the changes in metabolites, so as to further understand the physiological state of microorganisms (Ye et al., 2022; Meng et al., 2023). In the present study, the metabolic profile of *R. solani* induced by PA was investigated by widely targeted metabolomics. A comprehensive analysis of metabolites changes was performed, and the regulation of the metabolic network that may contribute to proliferation or reduction of fungal cells was annotated. The results obtained provide a foundation for further identification of the possible mechanisms of synergistic effects of autologous toxin and pathogen on *S. miltiorrhiza* as well as for the development of new strategies to control the occurrence of replantation diseases.

## 2 Materials and methods

### 2.1 Fungal strain

*R. solani* (GDMCC 3.700) was purchased from Guangdong Provincial Microbial Species Preservation Center, China. The fungal strain was transferred to potato dextrose agar (PDA) (Solarbio, Beijing, China) medium and incubated at 28°C in dark.

### 2.2 Effect of PA on colony growth and biomass of *Rhizoctonia solani*

Modified Potato Dextrose Agar (PDA) plates containing PA at final concentrations of 0.1, 1, 10, 100 and 500 mg·L^−1^ were employed for determining the effect of PA on *R. solani* colony growth. The PA solution was prepared in trace methanol and filtered with 0.22-µm membrane. PDA plates containing the same amount of methanol were used as control. Each treatment set up 4 repetitions. Under aseptic conditions, *R. solani* was inoculated (6 mm of activated fungal colony) onto the center of the modified PDA plates and incubated at 28°C in the dark. The colony diameters of each treatment group at 24,48,72 and 96 h after inoculation were measured by the cross method (measuring and calculating the average value of two mutually perpendicular diameters passing through the colony growth center).

To ascertain the effect of PA on fungal biomass, potato dextrose broth (PDB) (Solarbio, Beijing, China) supplemented with PA at a final concentration of 0 (control), 0.1, 1, 10, 100, and 500 mg·L^−1^ was used. Each treatment comprised three replicates. A certain amount of *R. solani* was suspended into sterile water and vortexed, and 100 μL of the fungal suspension were inoculated into PDB and incubated for 6 days at 120 rpm and 28°C. Then, the culture broth was centrifuged for 10 min at 10,000 × g and 4°C, the precipitate was collected, and the fresh weight of the mycelium was measured.

### 2.3 Effect of PA on intracellular polysaccharides and total fungal protein content in *Rhizoctonia solani*

The *R. solani* mycelia were washed twice with cold PBS and ground in liquid nitrogen. Then, 500 μL of filamentous fungal protein extract (Solarbio, Beijing, China) and 2 μL of protease inhibitor mixture were added to 100 mg of the fungal cells and shaken for 40 min at 4°C. Subsequently, the mixture was centrifuged for 10 min at 15,000 × g and 4°C, the supernatant was collected, and the fungal protein concentration was determined by BCA (bicinchoninic acid) method.

To ascertain the effect of PA on intracellular polysaccharides content in *R. solani*, 50 mg of freeze-dried fungal mycelia were added to an appropriate amount of distilled water and subjected to ultrasonic extraction for 31 min at 77°C and 335 W. The obtained extract was centrifuged for 10 min at 6000 rpm and the supernatant was mixed with 4 times volume of ethanol, allowed to stand overnight at 4°C, centrifuged, and vacuum freeze-dried. The intracellular polysaccharides content was determined by improved phenol-sulfuric acid method. In brief, the polysaccharides extract was mixed with 1 mL of phenol solution (5%) in a dry test tube, and 5 mL of concentrated sulfuric acid (98%) solution were added to the mixture and the optical density was observed at 490 nm (OD490).

### 2.4 Selection of optimal PA concentration and sample collection for metabolites extraction

The optimal PA concentration (100 mg·L^−1^) was selected based on the phenotypic data of co-culture of *R. solani* with different concentrations of PA. PDB medium was used as control group (CK). The group inoculated with Fungi without adding phthalic acid was described as Fungi, while the group supplemented with PA (100mg·L^-1^) and *R. solani* were described as PA-fungi. After incubation for 6 days, the culture was immediately frozen at −80°C after liquid nitrogen quenching for metabolites extraction.

### 2.5 Metabolites extraction

The samples frozen at −80°C were thawed and vortexed for 30 s. Then, the samples were frozen again overnight at −80°C in a refrigerator and vacuum freeze-dried. A total of 50 mg of the sample was weighed using an electronic balance (MS105DM) and mixed with 1200 μL of 70% methanol containing the internal standard extract. Subsequently, steel beads were added to the mixture and vortexed for 15 min, and then centrifuged for 3 min at 12,000 rpm and 4°C. The supernatant was collected, filtered through a microporous filter membrane (0.22-μm pore size), and analyzed by ultra-performance liquid chromatography electrospray ionization tandem mass spectrometry (UPLC-ESI-MS/MS) (Xiao et al., 2021).

### 2.6 UPLC-ESI-MS/MS

The sample extracts were analyzed using an UPLC-ESI-MS/MS system (UPLC, ExionLC™ AD; https://sciex.com.cn/) and tandem mass spectrometry system (https://sciex.com.cn/). The analytical conditions were as follows: column, Agilent SB-C18 (1.8 µm, 2.1 mm × 100 mm); mobile phase, ultrapure water (with 0.1% formic acid) as the aqueous phase and acetonitrile (with 0.1% formic acid) as the organic phase. The gradient program was as follows: water/acetonitrile, 95:5 (v/v) at 0 min, 5:95 (v/v) at 9.0 min, 5:95 (v/v) at 10.0 min, 95:5 (v/v) at 11.1 min, and 95:5 (v/v) at 14.0 min; flow rate, 0.35 mL min-1; column temperature, 40 C; and injection volume, 2 μL. The ESI source operation parameters were as follows: source temperature, 500 C; ion spray voltage (IS), 5500 V (positive ion mode)/−4500 V (negative ion mode); ion source gas I (GSI), gas II (GSII), and curtain gas (CUR) were set at 50, 60, and 25 psi, respectively; and collision-activated dissociation (CAD), high. For QQQ scan, MRM mode was employed and collision gas (nitrogen) was set to medium.

### 2.7 Qualitative and quantitative analysis of metabolites

Based on the self-built database MWDB (Metware database), the samples were characterized according to the secondary spectrum information (Yang et al., 2019; Xiao et al., 2021). The isotope signal and repeated signals containing K^+^ ions, Na^+^ ions, NH4^+^ ions, and fragment ions of other large molecular weight substances were removed during the analysis. Metabolites quantification was performed using MRM mode of triple quadrupole mass spectrometry (Nie et al., 2021). After obtaining the metabolic profiling data of different samples, the peak areas of all the chromatographic peaks were integrated, and the mass spectrum peaks of the same metabolite in different samples were corrected by MultiQuant software (AB SCIEX, Concord, Ontario, Canada) (Zhu et al., 2018). The peak area represents the relative content of the corresponding substance.

### 2.8 Data analysis

Through principal component analysis (PCA) of the samples (including quality control (QC) samples), the variability of the metabolites between and within the groups was preliminarily determined. Then, the original data of metabolites were processed by orthogonal partial least squares discriminant analysis (OPLS-DA), and the MetaboAnalystR package OPLSR.Anal function in R software was used for analysis to further show the differences between groups (Thévenot et al., 2015). Based on the variable importance in projection (VIP) obtained from the OPLS-DA model, the differential metabolites between groups (VIP > 1) were preliminarily screened. Subsequently, the selected differential metabolites (fold change (FC) ≥ 2 and ≤ 0.5) were further assessed by combining the FC values of univariate analysis-FC analysis. The metabolites content data were processed by unit variance (UV) scaling (see Appendix for specific formula). Heat maps were constructed by Complex Heatmap package of R software, and hierarchical cluster analysis (HCA) was performed on the metabolites accumulation patterns among different samples. The relative contents of all the differential metabolites identified in all the groups according to the screening criteria were subjected to UV scaling (see Appendix for specific formula), followed by K-Means cluster analysis. The Kyoto Encyclopedia of Genes and Genomes (KEGG) database (http://www.kegg.jp/kegg/compound/) was used to perform functional annotation and enrichment analysis of differential metabolites to determine the metabolic pathways that are highly correlated with the phenotypic changes of *R. solani*.

## 3 Results

### 3.1 Effects of PA on the colony growth and biomass of *Rhizoctonia solani*

**Figure 1:**
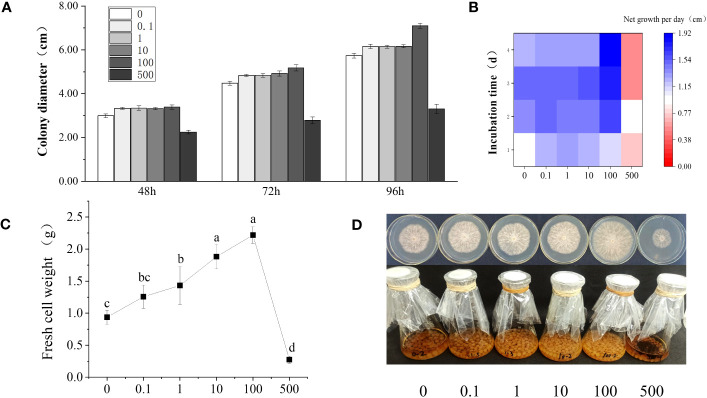
Changes in *R. solani* growth under different PA concentrations. **(A)** Colony diameter under different PA treatments (0–500 mg·L^−1^) at 48, 72, and 96 h; **(B)** Net mycelial growth per day under different PA treatments (0–500 mg·L^−1^) for 1–4 days; **(C)** Fresh weight of the mycelia under different PA treatments (0–500 mg·L^−1^) for 6 days; **(D)** Fungal phenotypic differences under different PA treatments (0–500 mg·L^−1^). The abscissa in (a–d) shows the PA concentration (mg·L^−1^).

In order to explore the effect of phthalic acid on the growth of *R. solani*, the *R. solani* colony diameter was determined at 48, 72, and 96 h, and the net mycelial growth under 0, 0.1, 1, 10, 100, and 500 mg·L^−1^ PA stress was measured each day (**Figures 1A, B**). The results showed that the *R. solani* growth first increased and then decreased with the increasing PA concentration. When compared with the control group (without PA addition), the net growth of *R. solani* on modified PDA plate containing 0.1–10 mg·L^−1^ PA presented almost no difference, and the colony diameter slightly increased at 48, 72, and 96 h. In the presence of 100 mg·L^−1^ PA, the *R. solani* colony diameter was 3.40, 5.18, and 7.10 cm at 48, 72, and 96 h, respectively, and the net mycelial growth per day was 1.65–1.92 cm, which was significantly higher than those noted in the control and other treatment groups (**Figures 1A, B**). The biomass of *R. solani* gradually increased with the increase in PA concentration, and reached the maximum (2.22 g) at 100 mg·L^−1^ PA, indicating that 100 mg·L^−1^ PA could significantly promote *R. solani* colony growth (**Figure 1C**). At the highest concentration (500 mg·L^−1^), PA showed a significant inhibitory effect on the colony growth and fungal biomass of *R. solani*. **Figure 1D** presents the *R. solani* phenotypic variations in different groups.

### 3.2 Effects of PA on intracellular polysaccharides and total protein contents in *Rhizoctonia solani*

**Figure 2:**
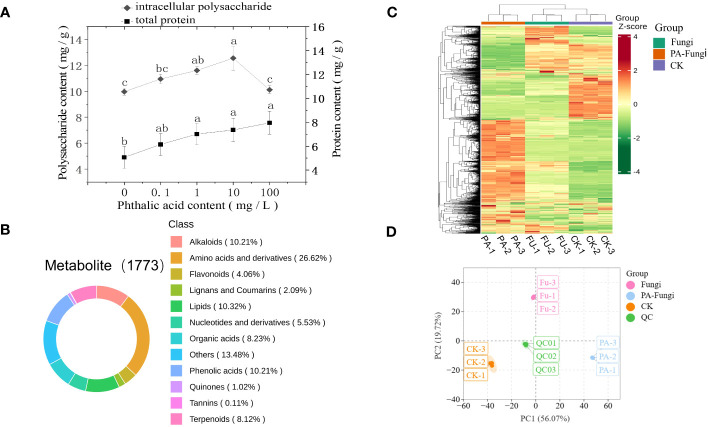
**(A)** Intracellular polysaccharides and total protein contents in *R. solani* under different PA treatments; **(B)** Classification of 1773 metabolites detected in the samples; **(C)** HCA of the metabolites; **(D)** Score plot of PCA.

As shown in **Figure 2A**, the total protein content in *R. solani* gradually increased with increasing PA concentration (0.1–100 mg·L^−1^ PA), presenting a certain dose effect, and reached the maximum at 100 mg·L^−1^ PA, indicating that a certain concentration range of PA significantly promoted the total protein accumulation in *R. solani*. When compared with the control group, the intracellular polysaccharides content in *R. solani* exhibited varying degrees of increase with increasing PA concentration (0.1–100 mg·L^−1^ PA), and was significantly higher under 10 mg·L^−1^ PA (**Figure 2A**), implying that 10 mg·L^−1^ PA can significantly promote intracellular polysaccharides accumulation in *R. solani*.

### 3.3 Overview of the metabolites of *Rhizoctonia solani*

To ensure data repeatability and reliability, QC samples were included for every 10 test samples during the analysis (Li and Song, 2019). The results showed a high overlap ratio of the total ion current (TIC) curves and same retention time and peak intensity of the QC samples, indicating that the test results were highly reliable (**Supplement Figure 1**). A total of 1773 metabolites, including primary and secondary metabolites, were obtained from nine samples by widely targeted metabolomics analysis. Among them, amino acids and their derivatives, other classes, lipids, phenolic acids, alkaloids, organic acids, terpenoids, nucleotides and their derivatives, flavonoids, lignin and coumarin, quinones, and tannin accounted for 26.62%, 13.48%, 10.32%, 10.21%, 10.21%, 8.23%, 8.12%, 5.53%, 4.06%, 2.09%, 1.02%, and 0.11%, respectively (**Figure 2B**).

The metabolites accumulation patterns in the *R. solani* fermentation broth under different PA treatments were visualized through heatmap and HCA. The heat map showed that some metabolites of *R. solani* were downregulated, whereas some were upregulated, when cultured with exogenous PA, indicating that the metabolic process of *R. solani* significantly varied with the addition of exogenous PA (**Figure 2C**).

### 3.4 Principal component analysis and orthogonal partial least squares-discriminant analysis

The PCA was performed with the QC samples and all treatment samples to understand the differences within and between the groups (**Figure 2D**) (Nie et al., 2021). The results showed only slight variation between each treatment sample, and a trend of separation was evident between the different treatment groups, suggesting that exogenous PA addition induced significant metabolic variations in *R. solani*, corresponding to the changes in colony growth and fungal biomass.

**Figure 3:**
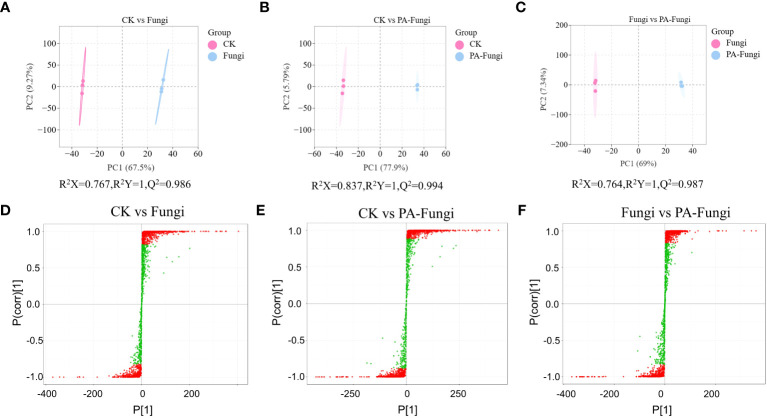
Scores of the OPLS-DA model. **(A)** CK vs.Fungi; **(B)** CK vs.PA-Fungi; **(C)** Fungi vs.PA-Fungi; S-plot of the OPLS-DA model with **(D)** CK vs.Fungi; **(E)** CK vs.PA-Fungi; **(F)** Fungi vs.PA-Fungi. R2Y scores and Q2 values represent the interpretation rate of the model to the Y matrix and the prediction ability of the model, respectively. Q2 > 0.5 indicates that the model is effective; Q2 > 0.9 denotes that the model is excellent.

Pairwise comparisons were achieved using the OPLS-DA model, and the score plots demonstrated that the R2X scores were higher than 0.76, while the R2Y scores and Q2 values were higher than 0.98 in CK vs. Fungi, CK vs. PA-Fungi, and Fungi vs. PA-Fungi samples (**Figures 3A–C**), confirming that the differential metabolites significantly responded to PA treatment. Furthermore, the differences in the metabolites expression levels between two groups were visualized through the S-plot of OPLS-DA (**Figures 3D–F**).

### 3.5 Identification of differential metabolites

**Figure 4:**
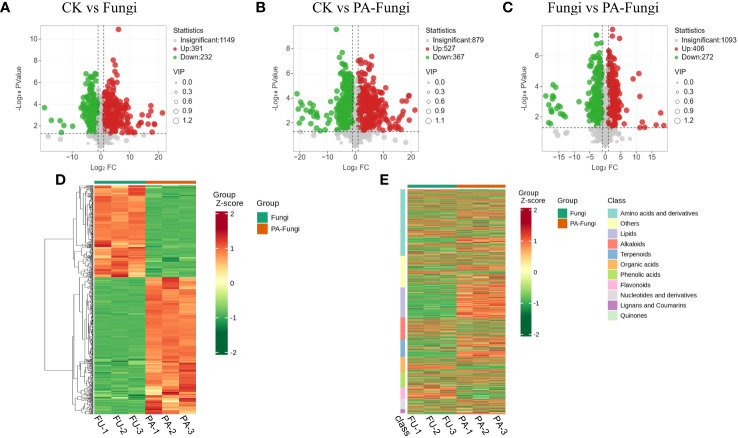
Volcano diagrams of differential metabolites in different pairwise comparisons. **(A)** CK vs. Fungi; **(B)** CK vs. PA-Fungi; **(C)** Fungi vs. PA-Fungi; **(D)** Cluster heat map of differential metabolites of Fungi vs. PA-Fungi; **(E)** Cluster heat map of differential metabolites (classified by component) of Fungi vs. PA-Fungi.

The differential metabolites were screened by univariate analysis (FC/P) and OPLS-DA using the screening criteria |log2FC| > 1, VIP > 1, and P < 0.05 for significantly different metabolites. The difference in the expression level of the metabolites between the two sample groups was visualized through a volcano plot (**Figures 4A–C**). As shown in **Table 1**, the number of upregulated metabolites in all pairwise comparisons was significantly higher than that of downregulated metabolites, and the number of upregulated and downregulated metabolites in CK vs. PA-Fungi was higher than that in CK vs. Fungi. These results indicated that co-culture with PA enhanced metabolism and activated the key physiological and metabolic activities related to proliferation in *R. solani*, leading to higher consumption of medium components and production of new metabolites.

**Table 1:** Statistics of the number of differential metabolites.

Group name	All sig diff	Down regulated	Up regulated
CK vs Fungi	623	232	391
CK vs PA-Fungi	894	367	527
Fungi vs PA-Fungi	678	272	406

**Figure 5:**
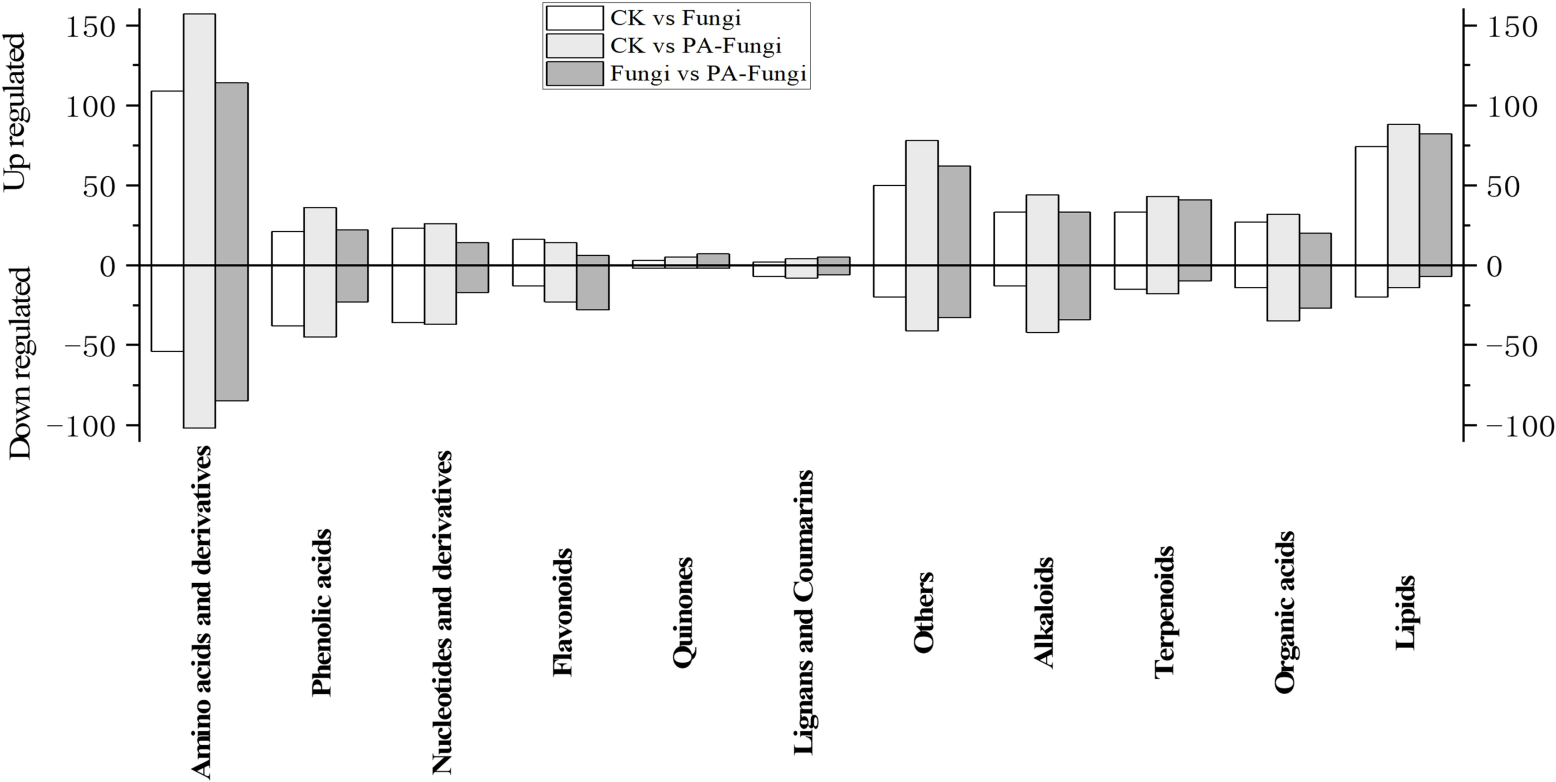
Column diagram of differential metabolites (classified by component) between groups (pairwise comparison).

To further reveal the changes in metabolites under PA treatment, the differential metabolites of Fungi vs. PA-Fungi were quantified using heat map (**Figure 4D**). The metabolite concentration was converted to log10 changes to reflect the differential metabolite (e.g., presented as green and red shadows in the heat map). The differential metabolites of the two groups were significantly clustered into two different color slices, indicating that there were substantial metabolic differences between PA-treated and untreated *R. solani*. As shown in **Figure 4E**, the differentially regulated metabolites were clustered into 11 categories, mainly concentrated in amino acids and their derivatives, other classes, lipids, and alkaloids. The contents of amino acids and their derivatives, lipids, and terpenoids in the PA-treated group were significantly increased, and the content of organic acids decreased significantly, indicating that these components are likely to be involved in the proliferation of *R. solani* (**Figure 5**).

**Figure 6:**
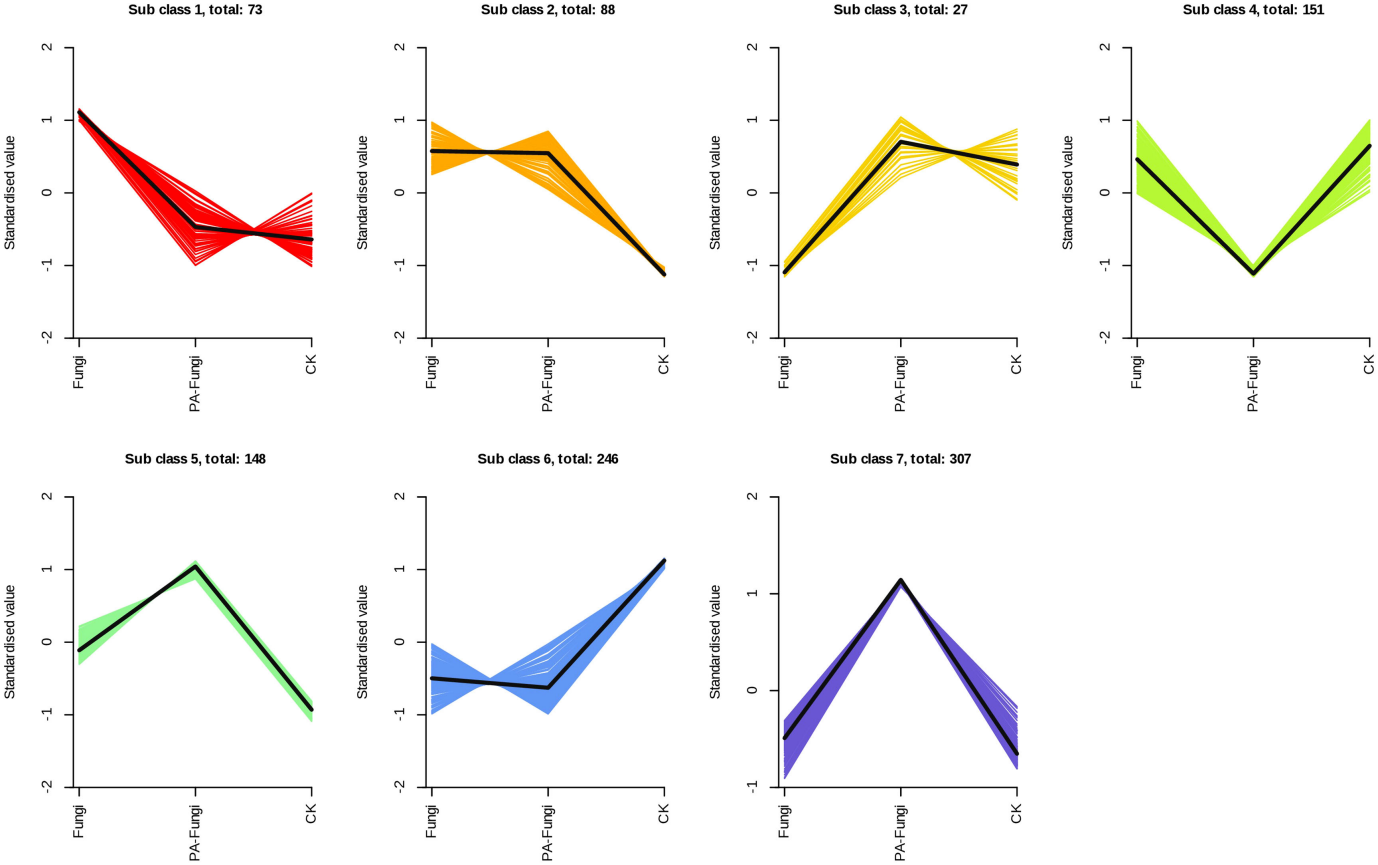
Analysis of the relative changes in the contents of differential metabolites in various groups. Note: Abscissa represents the sample grouping, ordinate denotes the normalized relative content of metabolites, sub-class indicates the metabolite category number with the same trend, and total represents the number of metabolites in this category.

The K-Means cluster analysis was used to explore the variations in the differential metabolites between the groups (Li et al., 2022) (**Figure 6**). The results showed that the variations could be divided into seven subcategories containing 73, 88, 27, 151, 148, 246, and 307 metabolites, respectively. When compared with the blank medium, groups 5 and 7 increased after *R. solani* inoculation, and the contents continued to increase after addition of exogenous PA. On the contrary, groups 4 and 6 decreased after *R. solani* inoculation, and the contents continued to decrease after PA treatment. Through the change trend of these differential metabolites, it can be seen that after the addition of PA, the basic metabolic pathway of *R. solani* was strengthened, and a small number of new physiological functions were also activated.

### 3.6 KEGG annotation and enrichment analysis of differential metabolites

**Figure 7:**
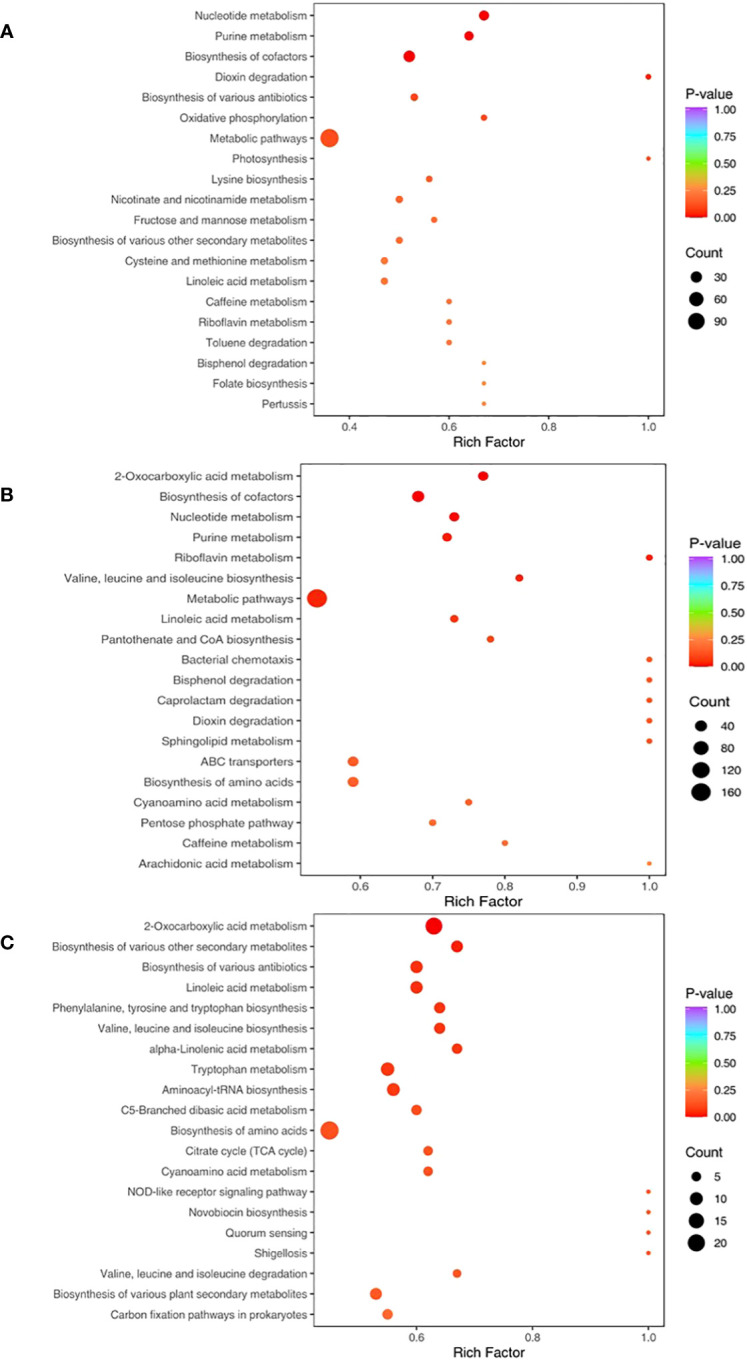
KEGG Enrichment map of differential metabolites. **(A)** CK vs. Fungi; **(B)** CK vs. PA-Fungi; **(C)** Fungi vs. PA-Fungi.

The relative metabolic pathways according to the results of KEGG annotation and enrichment are shown in **Figure 7**. When compared with CK, Fungi and PA-Fungi presented significant changes in nucleotide metabolism, purine metabolism, and cofactor bio-synthesis. Among them, Fungi exhibited significant changes in the biosynthesis of multiple antibiotics, oxidative phosphorylation, and lysine biosynthesis, whereas PA-Fungi presented significant alterations in 2-oxocarboxylic acid metabolism, valine, leucine and isoleucine biosynthesis, pantothenate and CoA biosynthesis, and sphingolipid metabolism. Further comparison between Fungi and PA-Fungi revealed 16 significantly enriched metabolic pathways. As shown in **Table 2**, of these 16 significantly differential metabolic pathways, 4, 3, 2, and 2 were related to amino acid metabolic pathways, biosynthesis of secondary metabolites, lipid metabolism, and glucose metabolic pathways, respectively. It is well known that the three major nutrients, namely, amino acids, lipids, and sugars, are closely related to energy metabolism, growth, and reproduction of cells. Thus, these enriched differential metabolic pathways indicated the PA-induced changes in the metabolic activities of *R. solani* and the possible mechanisms related to its proliferation.

**Table 2:** Analysis of differential metabolic pathways between Fungi and PA-Fungi group.

Category	Differential metabolic pathways
amino acid metabolism	Phenylalanine, tyrosine and tryptophan biosynthesis, Valine, leucine and isoleucine biosynthesis, Tryptophan metabolism, Valine, leucine and isoleucine degradation
metabolism of other amino acids	Cyanoamino acid metabolism
lipid metabolism	Linoleic acid metabolism, alpha-Linolenic acid metabolism
carbohydrate metabolism	C5-Branched dibasic acid metabolism, TCA cycle
biosynthesis of other secondary metabolites	Biosynthesis of various other secondary metabolites, biosynthesis of various antibiotics, biosynthesis of various plant secondary metabolites
Global and overview maps	2-Oxocarboxylic acid metabolism, biosynthesis of amino acids
energy metabolism	Carbon fixation pathways in prokaryotes
Translation	Aminoacyl-tRNA biosynthesis

**Figure 8:**
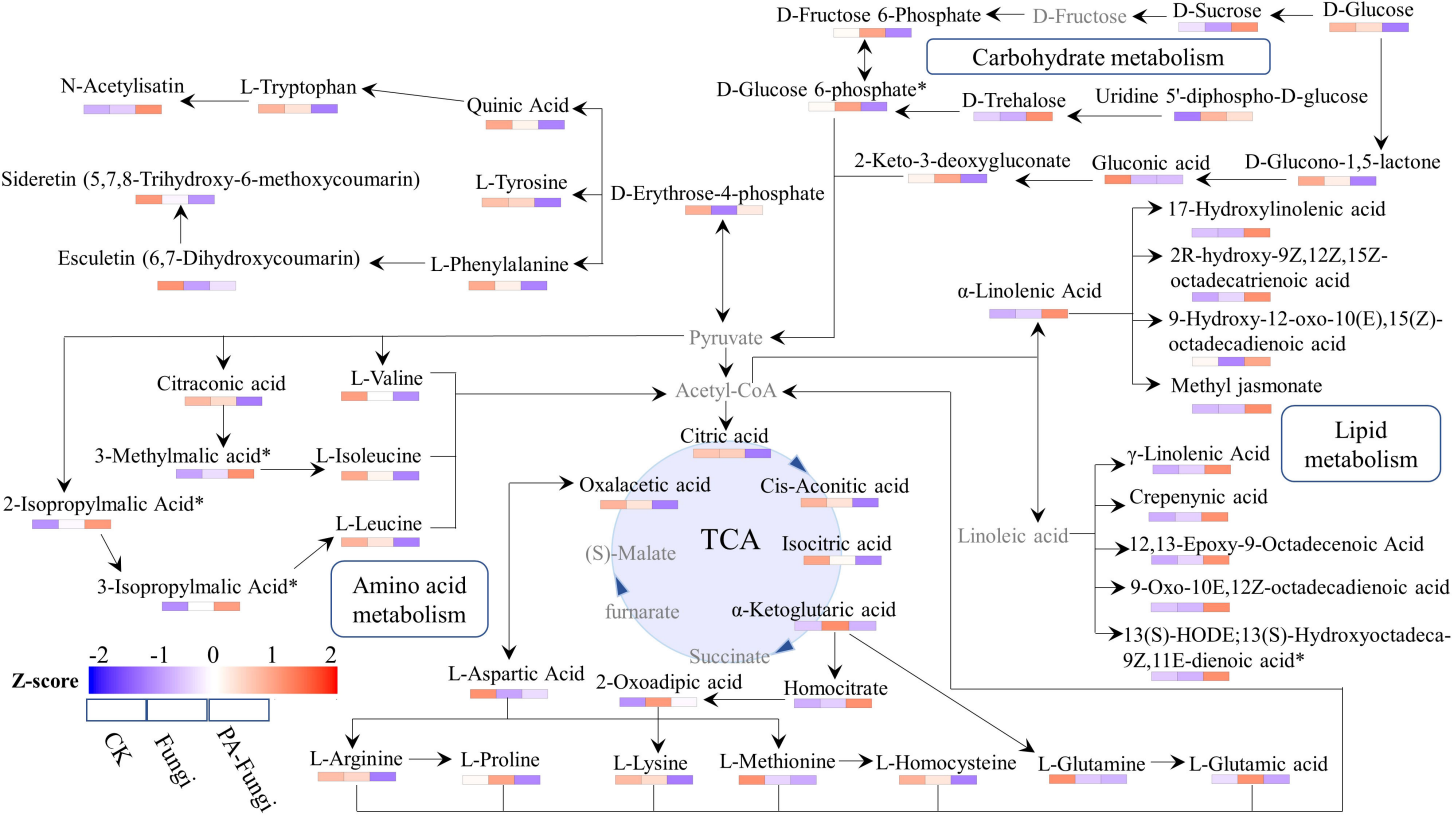
Metabolic network analysis of *R. solani* under PA stress. The proposed metabolic pathway was based on literature and network-based metabolic pathway database. Note: Undetected metabolites are presented in gray. The relative content of differential metabolites in various samples was standardized by Z-score, and the distribution of each differential metabolite in each group was spontaneously estimated by color.

To fully understand the changes in the metabolites of *R. solani* under PA stress, we proposed a metabolic pathway based on literature and network-based metabolic pathway database (Li et al., 2021). The main known pathways include tricarboxylic acid (TCA) cycle, linoleic acid metabolism, valine, leucine and isoleucine biosynthesis and degradation, α-linolenic acid metabolism, and amino acid biosynthesis (**Figure 8**). We identified four metabolites related to glycolysis pathway, including sucrose, glucose, glucose-6-phosphate, and fructose-6-phosphate. When compared with CK and Fungi groups, the sucrose content in PA-Fungi group was significantly increased, whereas glucose, glucose-6-phosphate, and fructose-6-phosphate contents were significantly reduced; besides, the contents of multiple monomeric amino acids such as valine, leucine, isoleucine, phenylalanine, and tryptophan were also significantly reduced in PA-Fungi group. Both amino acids and carbohydrates can enter the TCA cycle by generating acetyl-CoA, and α-linolenic acid and γ-linolenic acid are also synthesized from oxaloacetic acid through the TCA pathway. The contents of various long-chain fatty acids involved in linoleic acid and α-linolenic acid metabolic pathways were significantly increased in PA-Fungi group. These results indicated that PA treatment could effectively stimulate the basal metabolic response of *R. solani*, strengthen the consumption of amino acids and carbohydrate components in the medium for energy generation, and synthesize more lipids. In addition, the content of secondary metabolites such as esculetin and 5,7,8-trihydroxy-6-methoxycoumarin fluctuated in *R. solani* under PA treatment, indicating that the fungal secondary metabolism was also altered under PA stress.

## 4 Discussion

*Rhizoctonia solani*, a Basidiomycete, is a kind of necrotic fungus that causes damage to plants by producing plant cell wall degrading enzymes or extracellular toxins. The diversity of its host range and ability to maintain dormancy under adverse conditions further shape the strong lethality of *R. solani* (Senapati et al., 2022). At the same time, allelochemicals secreted by plants can be used as carbon and nitrogen sources by soil microorganisms or as signal substances to attract microorganisms (Castrillo et al., 2017; Li et al., 2017; Kelly et al., 2022), and organic acids have been proved to be the key plant metabolites with enhanced allelopathic activity (Dong et al., 2012; Wu et al., 2017; Wang et al., 2019). Therefore, in the present study, the growth inhibitory effects of PA on *R. solani* was examined. The results showed that PA promoted the growth of *R. solani* at low concentration and inhibited at high concentration. Our previous study found that PA accumulated in the rhizosphere of continuous cropping plants. The concentration of PA in the rhizosphere of *S. miltiorrhiza* for one year was 0.909 μg·g^−1^ (about 1.4 mg·L^-1^) (Jiao et al., 2022), which was 54.592% higher than that of non-continuous cropping *S. miltiorrhiza*. After continuous cropping for 3 years, the concentration of PA in the rhizosphere of *lily* was as high as 27.73 μg·g^-1^ (about 42 mg·L^-1^) (Wu et al., 2015). Combined with this study, it can be found that even if the concentration of PA in the rhizosphere of *S. miltiorrhiza* is low for one year of continuous cropping, it has reached the concentration that can cause the proliferation of *R. solani*. With the extension of continuous cropping years, the accumulation of PA increased, and the direct allelopathic effect on the growth and metabolism of *R.solani* was stronger. When the concentration of PA reaches a certain threshold, it will have an inhibitory effect on *R. solani*. Subsequently, the changes in the metabolites of *R. solani* after exogenous addition of PA were evaluated by widely targeted metabolomics. PCA and OPLS-DA revealed that exogenous PA significantly affected the metabolism of *R. solani*. Combined with the results of heat map analysis and K-means analysis of differential metabolites, it was found that most of the differential metabolites such as amino acids, lipids and organic acids may be related to the basic metabolic reactions of *R. solani*, and some new physiological metabolic pathways occur under acid treatment. Therefore, it can be reasonably speculated that phthalic acid enhances the basal metabolism of *R. solani* and activates some new secondary metabolic pathways.

Analysis of Fungi and PA-Fungi groups data showed that the number of upregulated and downregulated amino acids and their derivatives significantly increased, and the lipids were significantly upregulated in the PA stress group. It must be noted that the amino acid derivatives are synthesized by a series of reactions with amino acids as substrates. The increase in the content of amino acid derivatives in PA-treated *R. solani* indicated that PA significantly enhanced amino acid metabolism in the fungal cells, and possibly upregulated the expression of genes encoding enzymes related to amino acid metabolism. Amino acid synthesis and metabolism provide carbon and nitrogen sources and energy for cellular activities, as well as generate distinctive compounds, such as DNA, RNA, etc., required for cell growth through oxidative decarboxylation, deamination, and transamination (Vivancos et al., 2011). In the present study, the decrease in valine, leucine, iso-leucine, tyrosine, tryptophan, and phenylalanine contents in PA-Fungi group may be related to the enhancement of the physiological functions such as oxidative energy supply, protein synthesis, gluconeogenesis, and secondary metabolite synthesis (Yamamoto et al., 2017). The significant downregulation of proline and cysteine implied the activation of multiple bio-logical processes in *R. solani*, including signal transduction, antioxidant damage, and infection function (Mucip et al., 2015; Christgen and Becker, 2019). When compared with CK and Fungi, the amino acid monomer content was decreased and the contents of amino acids and its derivatives and total protein were increased in PA-Fungi group, which might be owing to the active resistance of fungal cells to PA stress and the enhancement of amino acid anabolism.

Furthermore, the contents of choline, ethanolamine, and fatty acids were significantly increased in PA-Fungi, suggesting that the metabolic function of phospholipids was enhanced in *R. solani* under PA stress. Phospholipids are important components of biofilms, and changes in their composition and content imply variations in the cell physiology and metabolic status (Patton-Vogt and de Kroon, 2020). Guo et al. found that organic acids have good affinity with cell membrane, which can dissociate the acid radical ions and hydrogen ions into cells and result in membrane structure destruction (Guo et al., 2022). It can be speculated that the inhibition of 500 mg·L^-1^ exogenous PA is related to this. Owing to its high polarity, accelerated hydrolysis of phospholipids may not be conducive to the penetration of strong polar substances into the cell membrane. The increase in the content of phospholipid hydrolysates observed in the present study could reduce the permeability of *R. solani* cell membrane to PA, thus protecting the cells. At the same time, accumulation of unsaturated fatty acids in the fungal cells may increase the content of permeability-reducing substances such as carotene, with fatty acids also exhibiting certain permeability-reducing effect, which can protect the fungal cells from free radical damage to some extent. In addition, high levels of fatty acids in the cells can produce a large amount of acetyl-CoA via β-oxidation, which is oxidized and decomposed by citric acid cycle in the mitochondria, providing immense energy for cellular activities. Thus, it can be inferred that the hydrolysis of phospholipids leads to an increase in lipids content, which causes a decrease in cell membrane permeability to PA and a series of biochemical reactions, providing sufficient impetus for the proliferation and infection of plant pathogenic fungi. Furthermore, the contents of α-linolenic acid and γ-linolenic acid in the PA treatment group were also significantly higher than those in the control group, indicating that PA stress may upregulate the expression of fungal lipoxygenase genes or strengthen the oxygenation reaction of unsaturated fatty acids. Previous studies have shown that oxygenated lipids derived from linoleic acid and linolenic acid protect important intracellular molecules such as DNA from reactive oxygen species attacks, and lipid oxide mediated signaling pathways are crucial to pathogen growth, reproduction, and host infection (Tsitsigiannis and Keller, 2007).

TCA cycle is the fundamental metabolic process in which cells metabolize nutrients and produce ATP (Ma et al., 2020). In the present study, when compared with the control, the con-tents of oxaloacetic acid, citric acid, cis-aconitic acid, isocitric acid, and α-ketoglutaric acid were significantly reduced in the PA treatment group, which could be attributed to the response of the fungal cells to PA stress. The conversion of citric acid to α-ketoglutaric acid is catalyzed by cis-aconitic acid and isocitrate dehydrogenase. The decrease in the contents of these organic acids, in turn, can regulate the synthesis of oxaloacetic acid and citric acid, enhance the activity of citric acid synthase, and accelerate citric acid cycle, thus providing more stress-resistant energy for the fungal cells. In addition, many intermediate products of the citric acid cycle are the precursor sources for the synthesis of biological macromolecules, can participate in other metabolic pathways through the citric acid cycle (Liu et al., 2020b), and are the central hubs linking metabolism and transformation of nutrients such as sugar, lipid, and protein. In the present study, with simultaneous amino acid metabolism and lipid metabolism, the *R. solani* cells could accumulate excessive amino acids and their derivatives and fatty acids, and accelerate decomposition through citric acid cycle, which could provide energy for cell growth repair as well as raw materials for cellular biosynthesis.

Biosynthesis of aminoacyl-tRNA is one of the essential steps for gene expression in cells. In the present study, PA treatment group showed an increase in the biosynthesis of secondary metabolites, which indicated that the fungal cells under PA stress did not maintain logarithmic growth, but entered a relatively stable mid-growth stage. It must be noted that substances that are not necessary for microbial growth and reproduction, such as antibiotics, toxins, etc., are predominantly produced during the mid-growth stage. PA is presumed to enhance the gene transcription and translation process in *R. solani*, leading to rapid growth and proliferation of cells, and causing quick transition of the fungal cells from the growth index stage to stable stage, as well as to the secondary metabolic stage.

**Figure 9:**
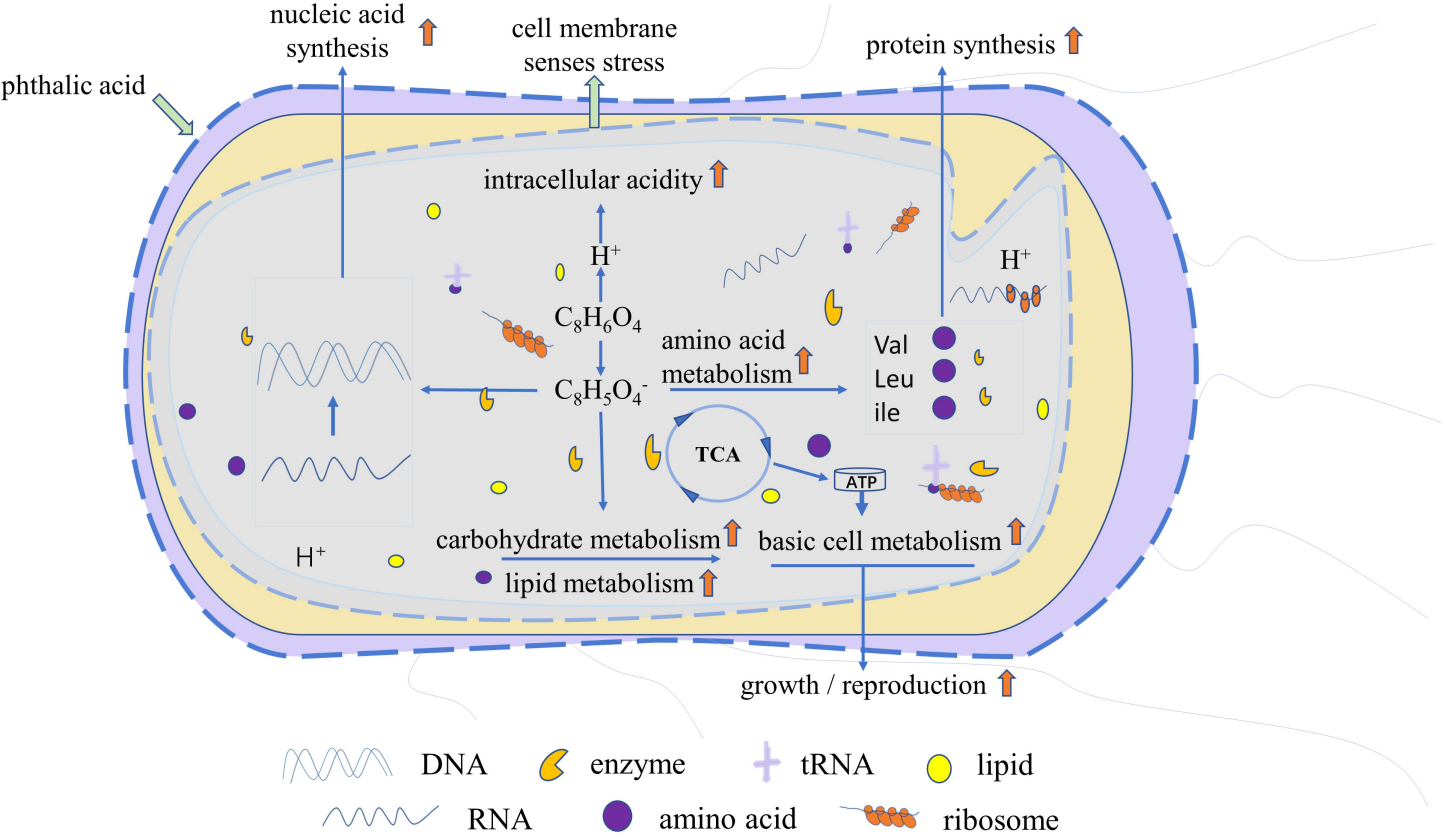
Proposed mechanism of promotion of *R. solani* proliferation by PA. Red upward arrow denotes increased amino acid metabolism, carbohydrate metabolism, lipid metabolism, basal cell metabolism, protein synthesis, and nucleic acid synthesis, and accelerated growth and reproduction.

The analysis of differential metabolites and metabolic pathways provided further insights into the interaction mechanism between *R. solani* and PA. Subsequently, we proposed a reasonable metabolic mechanism based on literature and network-based database (**Figure 9**) as follows: PA dissociates hydrogen ions and acid ions and enters the fungal cells. The fungal cells recognize the change in intracellular pH as an environmental stress, and initiate active defense strategies, including enhancement of amino acid metabolism, lipid biosynthesis, and carbohydrate metabolism. With the increase in nutrients consumption, the fungal cells also accumulate more amino acids and lipids as energy fuels for stress resistance. Following suppression of the increase in the PA concentration to toxic levels, the generated metabolic energy is utilized by the cells to promote synthesis of proteins, lipids, and nucleic acids, ultimately achieving rapid growth and proliferation. In the present study, some *R. solani* cells were found to exhibit earlier transition from primary metabolism to secondary metabolism. However, when the concentration of PA exceeds the physiological regulation range of *R. solani*, it has a strong inhibitory effect on its growth and reproduction.

The variations in the metabolites do not explain the exact mechanism by which PA promotes the proliferation of *R. solani*. Hence, in future, the biological functions of the proposed network must be characterized, such as identification of metabolite functions and protein changes.

## 5 Conclusion

This study revealed that PA, an allelochemical in the rhizosphere of *S. miltiorrhiza*, could exert a proliferation-driven effect on the growth of *R. solani*, a rhizosphere pathogen. Analysis of the metabolic profiles of *R. solani* before and after co-culture with PA revealed 1773 metabolites and 1040 differential metabolites, respectively. KEGG enrichment showed that these differential metabolites were involved in the biosynthesis and degradation of valine, leucine, and isoleucine, TCA cycle, linoleic acid and α-linolenic acid metabolism, amino acid metabolism, and other pathways. Subsequently, the variations of the differential metabolites in *R. solani* under PA stress were compre-hensively analyzed, and a metabolic network underlying the effect of PA on increasing the proliferation of *R. solani* was proposed. Enhancement of amino acid metabolism, significant increase in unsaturated fatty acid content, and enhancement of carbohydrate and energy metabolism were all related to the growth, proliferation, and active resistance of the fungal cells to PA stress. However, changes in metabolites do not explain the exact mechanism by which phthalates drive the proliferation of *R. solani*. In the future, more work needs to be done to characterize the biological functions of the proposed network, such as the identification of metabolite functions and protein changes. Through these, we will further understand the metabolic process of the synergistic interaction between pathogens and allelochemicals in the rhizosphere of continuous cropping *S. miltiorrhiza*, and lay a foundation for regulating the rhizosphere soil microecology and improving the planting level of *S. miltiorrhiza.*

## Data availability statement

The original contributions presented in the study are included in the article/**Supplementary Material**. Further inquiries can be directed to the corresponding author.

## Ethics statement

The manuscript presents research on animals that do not require ethical approval for their study.

## Author contributions

JJ: Conceptualization, Methodology, Writing – original draft. BZ: Investigation, Writing – review & editing. GY: Methodology, Writing – review & editing. XF: Data curation, Writing – original draft. XW: Resources, Writing – review & editing. LG: Methodology, Writing – review & editing. WL: Conceptualization, Formal Analysis, Funding acquisition, Methodology, Writing – original draft, Writing – review & editing.
