# Supplementary material for: Study on the metabolic process of phthalic acid driven proliferation of Rhizoctonia solani
Source: Front Plant Sci. 2023 Oct 11;14:1266916. doi: 10.3389/fpls.2023.1266916 (PMC10598758; doi:10.3389/fpls.2023.1266916)
Supplement: Supplementary file 1 [file Table_1.docx]

Supplementary Material

# Supplementary Figures


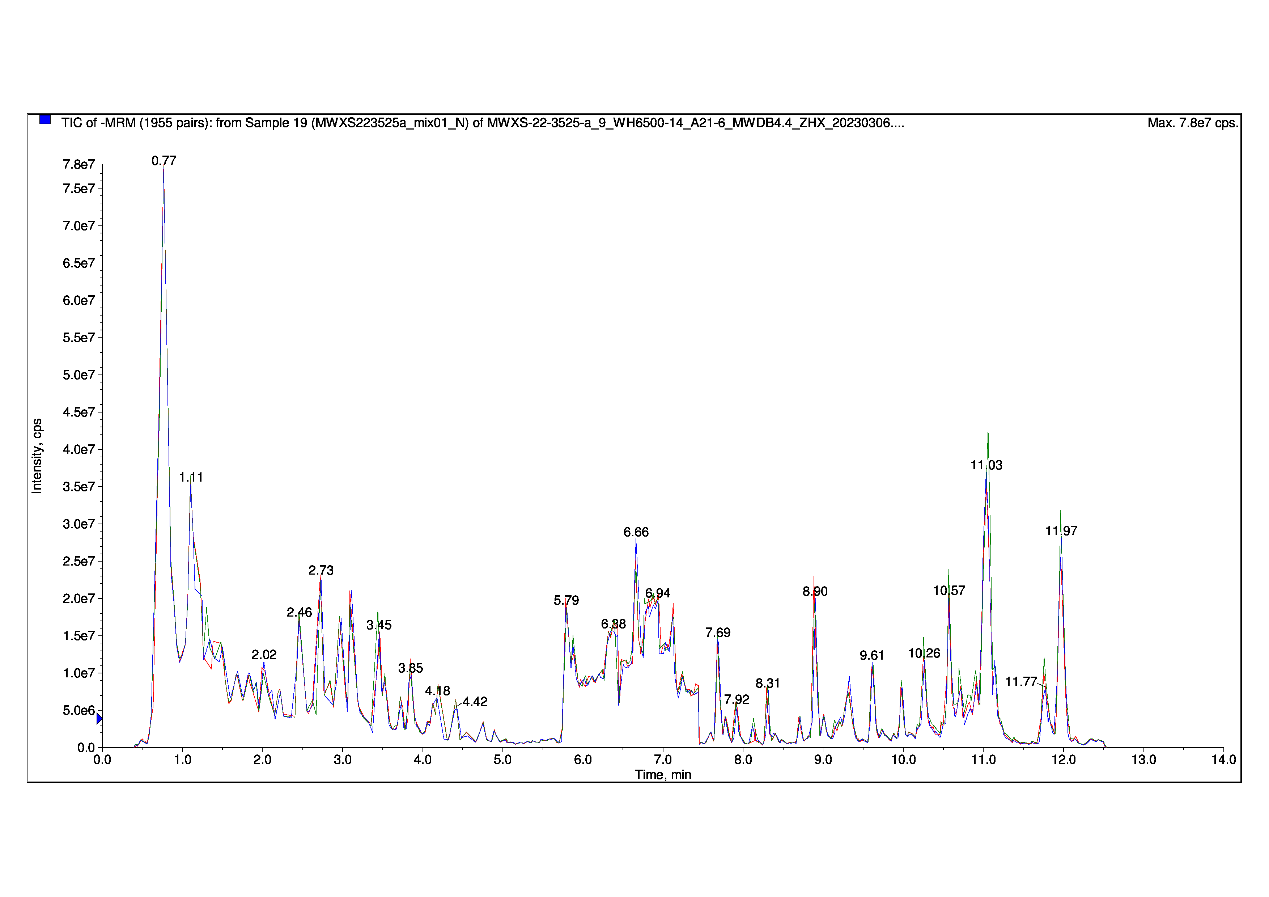

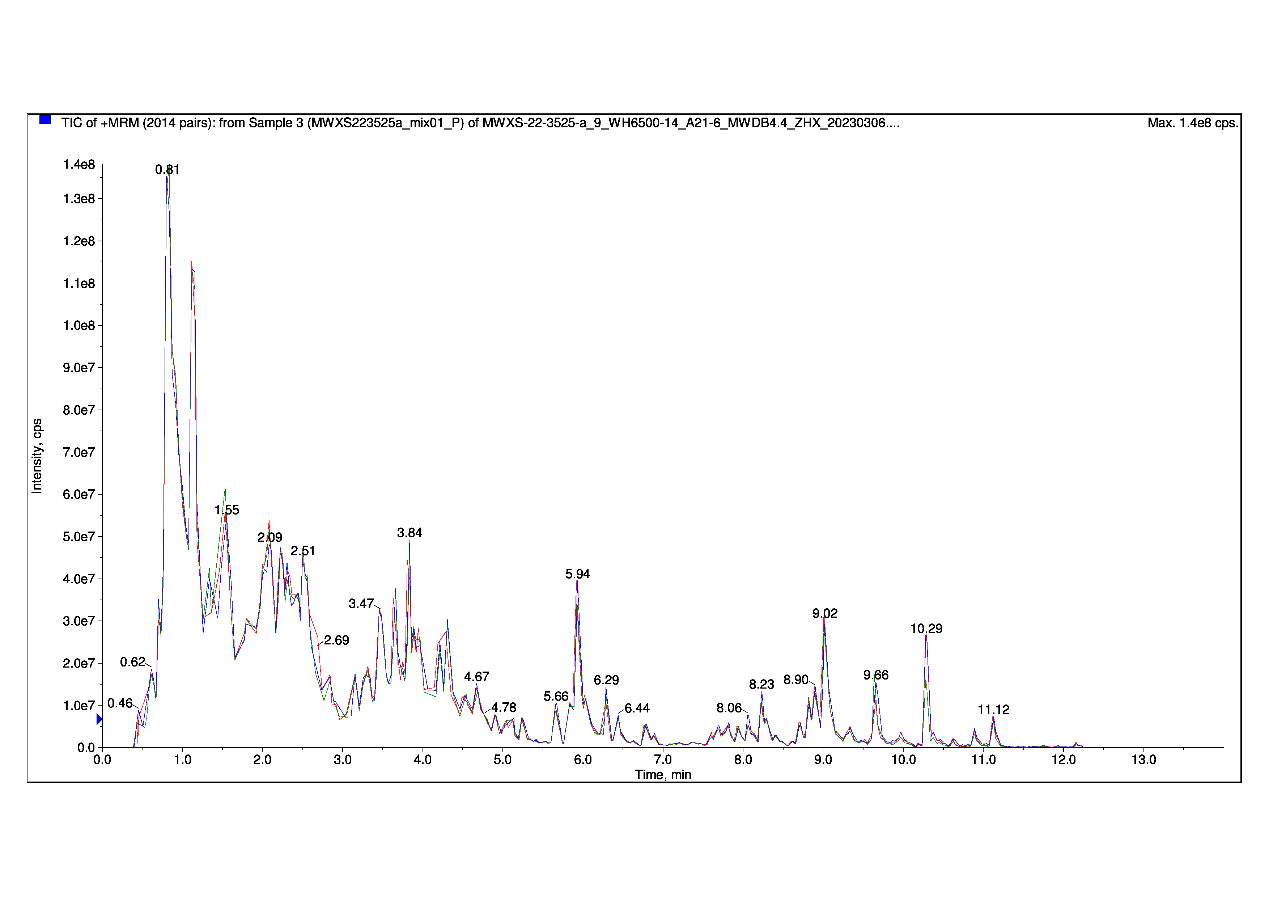


**Supplementary Figure 1.** Total ion current diagram of QC sample.
