# Supplementary material for: Study on the metabolic process of phthalic acid driven proliferation of Rhizoctonia solani
Source: Front Plant Sci. 2023 Oct 11;14:1266916. doi: 10.3389/fpls.2023.1266916 (PMC10598758; doi:10.3389/fpls.2023.1266916)
Supplement: Supplementary file 2 [file Table_2.docx]

1. The original data information corresponding to the pictures and tables in this article has been attached and uploaded, and the data files have been named according to the names of the corresponding pictures or tables.

2. The PCA analysis uses the basic function prcomp of the R language (version V3.5.1) to calculate the principal components, and then uses the ggplot2 package to plot.

3. OPLS-DA: First, the data is log2 + centralized, and then the OPLSR.Anal function of MetaboAnalystR package (version V1.0.1) in R language (version V3.5.1) is used for oplsda analysis, and then ggplot2 is used for drawing.

4. Kmeans: Use the cascadeKM function of the vegan package of R language (version V4.1.2) to do clustering, and then use the R basic function to draw the graph.
